# MicroRNA-126 enhances the biological function of endothelial progenitor cells under oxidative stress via PI3K/Akt/GSK3β and ERK1/2 signaling pathways

**DOI:** 10.17305/bjbms.2019.4493

**Published:** 2021-02

**Authors:** Qinqin Wu, Benling Qi, Xiaoyu Duan, Xiaoyan Ming, Fengqin Yan, Yingxia He, Xiaofen Bu, Shan Sun, Hong Zhu

**Affiliations:** 1 Department of Gerontology, The Central Hospital of Wuhan, Tongji Medical College, Huazhong University of Science and Technology, Wuhan, China; 2 Department of Geriatrics, Institute of Geriatrics, Union Hospital, Tongji Medical College, Huazhong University of Science and Technology, Wuhan, China

**Keywords:** Acute myocardial infarction, AMI, endothelial progenitor cells, EPC, PI3K/Akt/GSK3β, pathway, ERK1/2

## Abstract

Endothelial progenitor cell (EPC) transplantation is a safe and effective method to treat acute myocardial infarction (AMI). However, oxidative stress leads to the death of a large number of EPCs in the early stage of transplantation, severely weakening the therapeutic effect. Previous studies demonstrated that microRNAs regulate the biological function of EPCs. The aim of the current study was to investigate the effect of microRNA on the biological function of EPCs under oxidative stress. Quantitative reverse transcription PCR was performed to detect the expression of miR-126, miR-508-5p, miR-150, and miR-16 in EPCs from rats, among which miR-126 showed a relatively higher expression. Treatment with H_2_O_2_ decreased miR-126 expression in EPCs in a dose-dependent manner. EPCs were further transfected with miR-126 mimics or inhibitors, followed by H_2_O_2_ treatment. Overexpression of miR-126 enhanced the proliferation, migration, and tube formation of H_2_O_2_-treated EPCs. MiR-126 overexpression also inhibited reactive oxygen species and malondialdehyde levels and enhanced superoxide dismutase levels, as well as increased angiopoietin (Ang)1 expression and decreased Ang2 expression in H_2_O_2_-treated EPCs. Moreover, miR-126 participated in the regulation of phosphatidylinositol 3-kinase (PI3K)/protein kinase B (Akt)/glycogen synthase kinase 3β (GSK3β) and extracellular signal-regulated kinase 1/2 (ERK1/2) signaling in EPCs, where both pathways were activated after miR-126 overexpression in H_2_O_2_-treated EPCs. Overall, we showed that miR-126 promoted the biological function of EPCs under H_2_O_2_-induced oxidative stress by activating the PI3K/Akt/GSK3β and ERK1/2 signaling pathway, which may serve as a new therapeutic approach to treat AMI.

## INTRODUCTION

Acute myocardial infarction (AMI) is a common and critical acute cardiovascular disease. Great progress has been achieved in the treatment of AMI with drugs and surgical procedures, which partially restore blood supply to the infarcted area and prevent deterioration of cardiac function. However, the therapeutic effect is limited, and the mortality rate of AMI has not been satisfactorily controlled [1]. Recently, stem cell transplantation has increasingly gained attention in the treatment of myocardial infarction [2,3]. Among various types of stem cells, endothelial progenitor cells (EPCs) have consistently shown good efficacy in transplantation [4,5]. Many animal [6-8] and clinical [9] experiments have validated EPC transplantation as a safe and effective method to treat myocardial infarction. The procedure shows good application prospects by reducing the size of infarction, improving the wall motion of infarcted areas, increasing the left ventricular ejection fraction after infarction, and improving disease prognosis. Despite these advantages, post-AMI hypoxic-ischemic microenvironment and oxidative stress lead to the death of a large number of EPCs in the early transplantation stage, severely weakening the therapeutic effect [10,11]. We previously demonstrated that hydrogen peroxide (H_2_O_2_)-induced oxidative stress significantly affected the biological functions of EPCs such as secretion, proliferation, migration, and adhesion [12]. Similarly, augmented levels of post-AMI oxidative stress in the myocardium severely influence the biological function of transplanted EPCs [13]. Therefore, it is essential to improve the biological function of EPCs under oxidative stress to promote post-AMI myocardial repair.

MicroRNAs are small non-coding RNAs that act as post-transcriptional regulators of gene expression in animals and plants [14]. They actively participate in pathophysiological processes such as growth and development, hematopoietic processes, angiogenesis, cell proliferation, and apoptosis and are closely related to cardiovascular diseases [15,16]. Several microRNAs, such as miR-126 and miR-17, are specifically expressed in EPCs and regulate EPC proliferation, migration, angiogenesis, aging, and other EPC functions [17-19]. In addition, microRNAs enhanced the capacity of EPCs to repair heart injury [20]. However, the molecular mechanism of microRNAs underlying the regulation of EPCs is not clear.

Previous research has revealed that microRNAs influenced cellular functions by targeting phosphatidylinositol 3-kinase (PI3K)/protein kinase B (Akt) signaling [21-23]. This pathway is involved in regulating various biological processes and was shown to exert a protective effect on oxidative stress-induced EPC injury [24-26]. However, whether the microRNA-mediated regulation of EPC function is related to PI3K/Akt signaling remains unclear. In this study, based on the previous research and databases, the expression of miR-126, miR-508-5p, miR-150, and miR-16 was evaluated in EPCs [27-29]. Among them, miR-126 was detected with relatively high expression in EPCs. EPCs were stimulated with H_2_O_2_, which decreased the expression of miR-126 in a dose-dependent manner. To this end, miR-126 was overexpressed or inhibited to evaluate its regulatory effect on EPC function and PI3K/Akt signaling under oxidative stress.

## MATERIALS AND METHODS

### Cell extraction and culture

Four-week-old male Sprague Dawley rats were purchased from the Hubei Provincial Center for Disease Control and Prevention. Bone marrow-derived mononuclear cells were isolated from the rats through density gradient centrifugation using Ficoll-Hypaque (TBD, Tianjin, China). The harvested cells were seeded into 6-well plates pre-coated with fibronectin (5 μg/mL, Millipore, Boston, MA, USA) at 1 × 10^6^ cells/well with endothelial growth medium (EGM-2, LONZA, Basel, Switzerland). After 24 h of incubation at 37°C in an atmosphere with 5% CO_2_, the medium was changed and non-adherent cells were discarded. Passaging was performed when the confluence reached 70–80%, and the medium was changed every 2 days. Cell morphology was observed by fluorescence microscopy (Olympus, Japan) and spindle-shaped cells were identified as bone marrow-derived EPCs.

### Characterization of EPCs

CD133 and CD34, which are expressed in bone marrow-derived EPCs at the early stage, are characteristic molecular markers of EPCs [30,31]. The percentage of harvested CD133+ and CD34+ cells was detected by flow cytometry (Beckman, Coulter, Brea, CA, USA). Cells (1 × 10^6^) were centrifuged at 1000 × g for 5 min and resuspended in 300 μL of phosphate-buffered saline [PBS] (Bioswamp, Myhalic Biotechnology Co., Ltd., Wuhan, China) containing 10% fetal bovine serum (FBS) in a 1.5 mL centrifuge tube. Then, 6 μL of CD133-allophycocyanin (Abcam, Cambridge, UK) and 6 μL of CD34-fluorescein isothiocyanate [FITC] (eBioscience, CA, USA) were added. Thereafter, the cells were incubated in the dark for 1 h at 4°C. After two washes with pre-cooled PBS and centrifugation at 300 × g for 5 min, the cells were resuspended in 300 μL of flow cytometry buffer (BD bioscience, USA). The cells were analyzed and data were acquired using a flow cytometer (Beckman Coulter, Brea, CA, USA). Furthermore, EPCs were identified by double staining of 1,1’-dioctadecyl-3,3,3’,3’-tetramethylindocarbocyanine-labeled acetylated low-density lipoprotein (Dil-AcLDL, Molecular Probes, Invitrogen, Carlsbad, CA, USA) and FITC-labeled lectin from Ulex europaeus agglutinin-1 (FITC-UEA-1, Sigma, Missouri, USA) [32]. Cells in the logarithmic phase (1 × 10^6^ cells/mL) were cultured for 3 days, and adherent cells were incubated with Dil-AcLDL (24 μg/mL) for 1 h. The cells were then fixed in 4% paraformaldehyde for 10 min and counterstained with FITC-UEA-1 (10 μg/mL) for 1 h. Fluorescent images were acquired under an inverted fluorescence microscope (Olympus, Tokyo, Japan). The expression of miR-126, miR-508-5p, miR-150, and miR-16 in EPCs was measured by quantitative reverse transcription polymerase chain reaction (qRT-PCR), with miR-126 showing relatively high expression. Therefore, the relationship between miR-126 and the biological function of EPCs was explored in the subsequent experiments.

### Cell treatment

EPCs in the logarithmic phase were treated with H_2_O_2_ at different concentrations (200, 400, 600, 800, and 1000 μM). Cell viability and miR-126 expression were evaluated using cell counting kit-8 (CCK-8) assay and qRT-PCR, respectively. The optimal experimental concentration of H_2_O_2_ was selected to be 600 μM. EPCs were transfected with miR-126 mimics or inhibitors (Guangzhou RiboBio, Co., Ltd. Guangzhou, China), followed by H_2_O_2_ treatment for 6 h. The EPCs were divided into eight experimental groups based on treatment: control (CON, no treatment); H_2_O_2_ (treated with 600 μM H_2_O_2_); miR-126 mimic; miR-126 mimic negative control (mimic-NC); miR-126 inhibitor; miR-126 inhibitor negative control (inhibitor-NC); H_2_O_2_ + miR-126 mimic; and H_2_O_2_ + miR-126 inhibitor.

### qRT-PCR

Total RNA was extracted using Trizol reagent (Ambion, TX, USA) and DNA in the extracted RNA was eliminated using DNase I (Fermentas, Thermo Fisher, Massachusetts, USA). cDNA was synthesized from total RNA (500 ng) using the M-MuLV kit (TAKARA, Dalian, China). qRT-PCR was carried out using the SYBR Green PCR kit according to the manufacturer’s instructions. The primer sequences are as follows: miR-126-F, 5’-GGGCATTATTACTTTT-3’, miR-126-R, 5’-AACTGGTGTCGTGGAGTCGGC-3’; miR-508-5p-F, 5’-GGGTACTCCAGAGGGC-3’, miR-508-5p-R, 5’-AACTGGTGTCGTGGAGTGGC-3’; miR-150-F, 5’-GGG TCTCCCAACCCTTG-3’, miR-150-R, 5’-AACTGGTGTCG TGGAGTCGGC-3’; miR-16-F, GGGTAGCAGCACGTA AA-3’, miR-16-R, 5’-AACTGGTGTCGTGGAGTCGGC-3’; U6-F, 5’-CTCGCTTCGGCAGCACATATACT-3’, U6-R, 5’-ACGCTTCACGAATTTGCGTGTC-3’. U6 served as an internal control. The 2^−ΔΔCt^ method was utilized to calculate the relative expression level of miR-126 in EPCs treated with different concentrations of H_2_O_2_. The expression of miR-126, miR-508-5p, miR-150, and miR-16 in EPCs was calculated using the 2^−ΔCt^ method [33]. All experiments were performed in triplicate.

### CCK-8 assay

The CCK-8 (Bioswamp) assay was performed to evaluate cell viability according to the manufacturer’s instructions. Harvested cells were seeded into a 96-well plate at 3 × 10^3^ cells/well. The cells were treated with H_2_O_2_ at different concentrations and/or transfected with miR-126 mimics or inhibitors, and 10 μL of CCK-8 solution was added to each well. After 4 h of incubation, the absorbance of the wells was measured using a microplate reader (Thermo Scientific, USA) at 450 nm.

### Flow cytometry

Intracellular reactive oxygen species (ROS) levels and apoptosis were assessed by flow cytometry. For the intracellular ROS assay, the harvested cells at a concentration of 1 × 10^7^ cells/mL were mixed with the diluted DCFH-DA (Bioswamp) fluoroprobes. The cells were incubated for 20 min at 37°C with gentle shaking every 4 min to ensure sufficient contact with the probes. After three washes with a serum-free culture medium, the cells were collected and measured by flow cytometry. For the apoptosis assay, the Annexin V-FITC/propidium iodide (PI) assay (Bioswamp) was performed according to the manufacturer’s protocol. The harvested cells at a concentration of 5 × 10^5^ cells were resuspended in 200 μL of binding buffer, 10 μL of Annexin V-FITC, and 10 μL of PI. The cells were incubated for 30 min in the dark and subjected to flow cytometry.

### Transwell migration assay

Treated cells were cultured in serum-free EGM-2 for 24 h and digested with 0.25% trypsin. After washing with serum-free EGM-2, the cells were resuspended in EGM-2 supplemented with 1% FBS at 1 × 10^5^ cells/mL. Then, 0.5 mL of cells were added to the top Transwell chamber, while 0.75 mL of EGM-2 containing 10% FBS was added to the lower chamber. After 48 h of culture at 37°C, the cells were fixed with 4% formaldehyde for 10 min and stained with 0.5 % crystal violet for 30 min. Finally, the cells were observed under a microscope (Nikon, Japan).

### Tube formation assay

After transfection and treatment with H_2_O_2_ for 6 h, the EPCs were resuspended in EGM-2 supplemented with 10% FBS and seeded in a Matrigel-coated 96-well plate (CORNING, USA) at 2 × 10^5^ cells/mL. After 4 h of incubation at 37°C, the formation of capillary-like structures was photographed under an inverted fluorescence microscope (Olympus, Tokyo, Japan).

### Enzyme-linked immunosorbent assay (ELISA)

The activity of superoxidase dismutase (SOD) and level of malondialdehyde (MDA) in the supernatant of treated EPCs were determined using respective ELISA kits (Bioswamp) according to the manufacturer’s protocols.

### Western blot

Total proteins were extracted from EPCs using radioimmunoprecipitation assay lysis buffer (Bioswamp) supplemented with protease and phosphatase inhibitors. The proteins were quantified using a bicinchoninic acid assay kit (Bioswamp). The obtained proteins (20 μL) were separated using sodium dodecyl sulfate-polyacrylamide gel electrophoresis and transferred to polyvinylidene fluoride membranes (Millipore). The membranes were blocked with 5% skim milk for 2 h at room temperature and incubated overnight at 4°C with the following primary antibodies: PI3K (Abcam, 1:1000), Akt (Bioswamp, 1:1000), p-Akt (Bioswamp, 1:1000), glycogen synthase kinase 3β (GSK3β, Abcam, 1:5000), p-GSK3β (Abcam, 1:1000), extracellular signal-regulated kinase 1/2 (ERK1/2, Abcam, 1:1000), p-ERK1/2 (Abcam, 1:1000); caspase 3 (Bioswamp, 1:1000), angiopoietin (Ang)1 (Abcam, 1:500), Ang 2 (Abcam, 1:5000), and glyceraldehyde 3-phosphate dehydrogenase [GAPDH] (CST, 1:1000). After washing, the membranes were incubated with a goat anti-rabbit IgG secondary antibody (Bioswamp, 1:20000) at room temperature for 1 h. Immunoreactivity was visualized by colorimetric reaction using enhanced chemiluminescence substrate buffer (Millipore) using an automatic chemiluminescence analyzer (Tanon-5200, Shanghai, China). The band gray values were measured by TANON GIS software.

### Statistical analysis

Data are expressed as the mean ± standard deviation (SD). One-way analysis of variance followed by the least significant difference test was used to compare differences between groups using IBM SPSS Statistics for Windows, Version 19.0. (IBM Corp., Armonk, NY, USA). A value of *p* < 0.05 was considered statistically significant.

## RESULTS

### EPCs were successfully extracted and exhibited relatively high miR-126 expression

**FIGURE 1:**
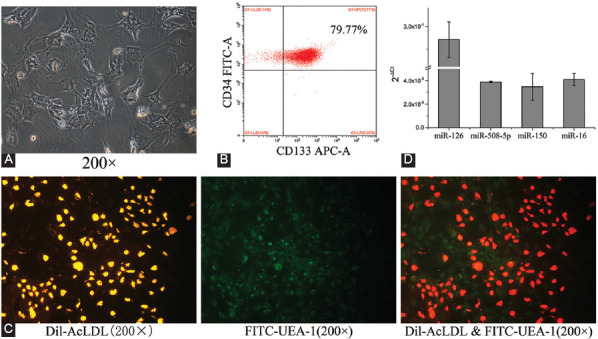
Characterization of EPCs. (A) Morphology of EPCs under a bright-field microscope. (B) Identification of the percentage of CD133+ and CD34+ EPCs. (C) Dil-AcLDL and FITC-UEA-1 double staining of EPCs. (D) Expression of miR-126, miR-508-5p, miR-150, and miR-16 in EPCs. Data are expressed as the mean ± standard deviation [SD] (n = 3). EPCs: Endothelial progenitor cells; Dil-AcLDL: 1,1’-dioctadecyl-3,3,3’,3’-tetramethylindocarbocyanine-labeled acetylated low-density lipoprotein; FITC: Fluorescein isothiocyanate; UEA-1: Ulex europaeus agglutinin 1.

To verify that EPCs have been successfully extracted, we visualized the typical morphology of EPCs using microscopy (Figure 1A). Flow cytometry was carried out to measure the percentage of CD133+ and CD34+ populations in the isolated cells. These markers are expressed in the early stage of bone marrow-derived EPCs. The results showed that the percentage of CD133+ and CD34+ cells was as high as 79.99% (Figure 1B). Meanwhile, positive staining was demonstrated for both Dil-AcLDL and FITC-UEA-1 (Figure 1C), confirming that the isolated cells were differentiating EPCs [34]. Furthermore, the expression of miR-126, miR-508-5p, miR-150, and miR-16 in EPCs was measured by qRT-PCR. The threshold cycle of miR-126 was lower than that of the other microRNAs (Figure 1D), demonstrating relatively high miR-126 expression in EPCs. Thus, the relationship between miR-126 and the biological function of EPCs was explored in subsequent experiments.

### Selection of H_2_O_2_ treatment concentration

**FIGURE 2:**
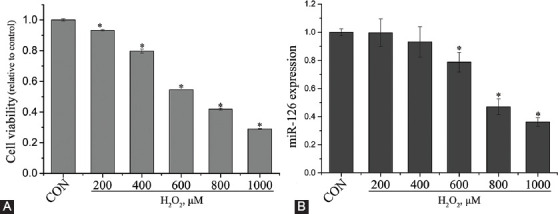
Selection of optimal hydrogen peroxide (H_2_O_2_) concentration. (A) Viability and (B) miR-126 expression of endothelial progenitor cells after treatment with H_2_O_2_ at different concentrations. Data are expressed as the mean ± standard deviation [SD] (n = 3), *p < 0.05 vs. control (CON).

The isolated EPCs were treated with H_2_O_2_ at 200, 400, 600, 800, and 1000 μM to select the optimal treatment concentration. CCK-8 and qRT-PCR were performed to evaluate cell viability and miR-126 expression, respectively. Figure 2 shows that the cell viability was decreased in a H_2_O_2_ dose-dependent manner, with significant differences compared with CON group (*p* < 0.05). At a concentration of 600 μM, H_2_O_2_ significantly downregulated miR-126 expression compared to that in control EPCs (*p* < 0.05). Thus, 600 μM H_2_O_2_ was chosen for the subsequent experiments.

### miR-126 expression

**FIGURE 3:**
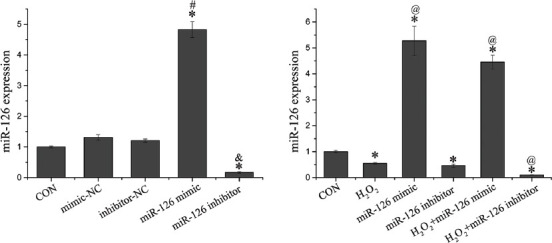
Expression of miR-126 in endothelial progenitor cells subjected to transfection and/or hydrogen peroxide (H_2_O_2_) treatment. Data are expressed as the mean ± standard deviation [SD] (n = 3), *,^#^,&, and ^@^ represent p < 0.05 vs. control (CON), mimic-negative control (NC), inhibitor-NC, and H_2_O_2_ , respectively.

After transfection and/or H_2_O_2_ treatment, the expression of miR-126 was measured by qRT-PCR (Figure 3). Compared to CON group, cells transfected with miR-126 mimics or inhibitors showed significantly higher or lower expression of miR-126 (*p* < 0.05), respectively. The expression of miR-126 among mimic-NC, inhibitor-NC, and CON groups showed no difference. Compared to H_2_O_2_ group, the expression of miR-126 in H_2_O_2_+miR-126 mimic group was upregulated (*p* < 0.05), while that in H_2_O_2_+miR-126 inhibitor group was downregulated (*p* < 0.05).

### Effect of miR-126 on the biological function of EPCs

**FIGURE 4:**
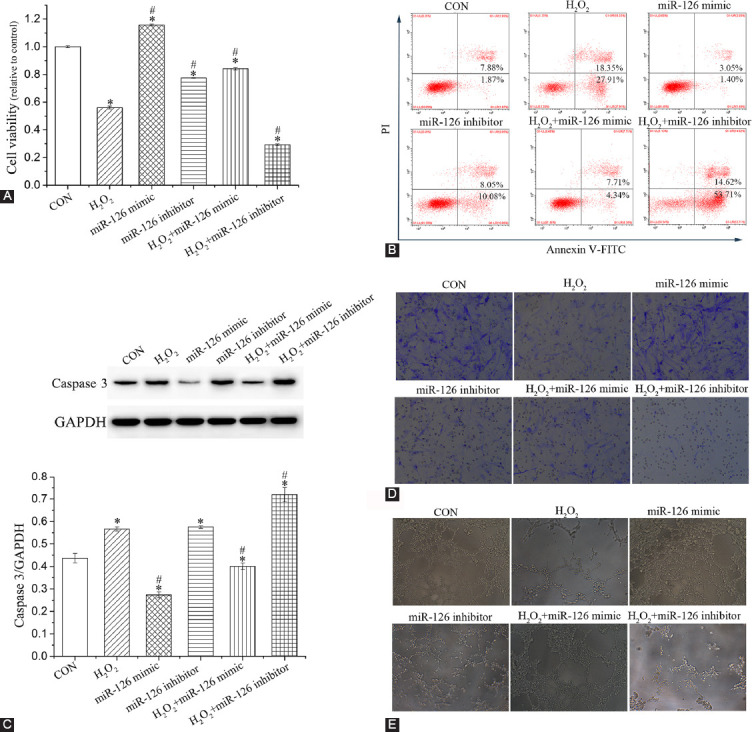
Evaluation of the biological function of EPCs. (A) EPC viability. (B) Percentage of EPC apoptosis. (C) Protein expression of caspase 3. (D) EPC migration. (E) Tube formation of EPCs. Data are expressed as the mean ± standard deviation [SD] (n = 3), * and ^#^ represent *p* < 0.05 vs. CON and H_2_O_2_, respectively. EPCs: Endothelial progenitor cells; GAPDH: Glyceraldehyde 3-phosphate dehydrogenase; CON: Control.

Evaluation of EPC function showed that H_2_O_2_ and miR-126 inhibitors significantly reduced EPC viability (*p* < 0.05). Compared to H_2_O_2_ group, cell viability was increased in H_2_O_2_+miR-126 mimic group (*p* < 0.05) but decreased in H_2_O_2_+miR-126 inhibitor group (*p* < 0.05; Figure 4A). As anticipated, the percentage of apoptosis showed the opposite trend as that of cell viability (Figure 4B). Moreover, the expression of the pro-apoptotic protein caspase 3 in H_2_O_2_ and miR-126 inhibitor groups was enhanced compared to that of control EPCs (*p* < 0.05; Figure 4C). Compared to H_2_O_2_ group, the expression of caspase 3 was reduced in H_2_O_2_+miR-126 mimic group (*p* < 0.05) but elevated in H_2_O_2_+miR-126 inhibitor group (*p* < 0.05). These results are consistent with those of apoptosis. Transwell migration and tube formation assays (Figure 4D and E) showed that H_2_O_2_ inhibited EPC migration and tube formation. MiR-126 inhibitors further contributed to this inhibition, while miR-126 mimics remarkably improved EPC migration and tube formation.

### Effect of miR-126 on ROS production, MDA level, and SOD activity

**FIGURE 5:**
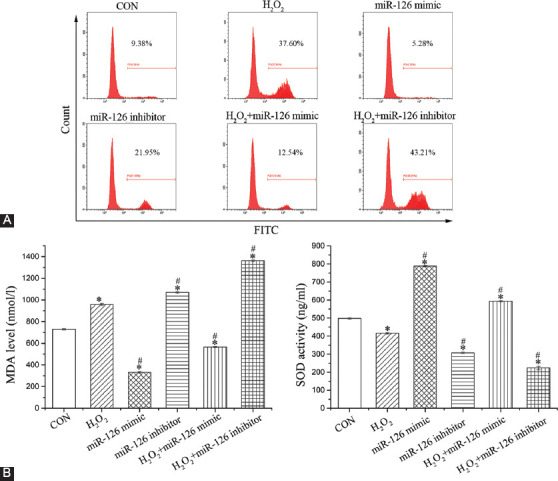
Assessment of oxidative stress-related indicators. (A) ROS production; (B) MDA level; and (C) SOD activity in EPCs. Data are expressed as the mean ± [SD] (n = 3), * and ^#^ represent *p* < 0.05 vs. CON and H_2_O_2_, respectively. ROS: Reactive oxygen species; MDA: Malondialdehyde; SOD: Super oxidase dismutase; EPCs: Endothelial progenitor cells; CON: Control.

As shown in Figure 5, compared with CON group, H_2_O_2_ and miR-126 inhibitor notably elevated the levels of ROS and MDA (*p* < 0.05), while significantly decreasing SOD activity (*p* < 0.05). In addition, compared to H_2_O_2_ group, ROS production and MDA activity were reduced in H_2_O_2_+miR-126 mimic group (*p* < 0.05), whereas SOD activity was significantly increased (*p* < 0.05). The H_2_O_2_+miR-126 inhibitor group showed the opposite trend (*p* < 0.05).

### Effect of miR-126 on the protein expression of Ang1 and Ang2

**FIGURE 6:**
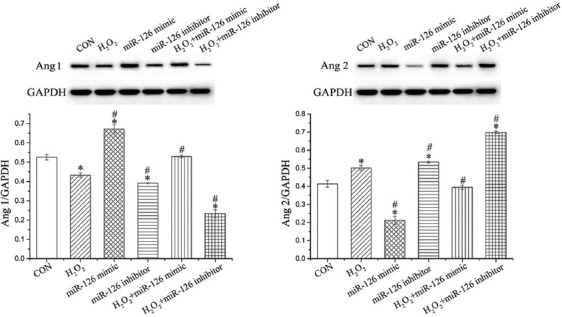
Expression of Ang1 and Ang2 in EPCs. Data are expressed as the mean ± standard deviation [SD] (n = 3), * and ^#^ represent *p* < 0.05 vs. CON and H_2_O_2_, respectively. Ang: Angiopoietin; EPCs: Endothelial progenitor cells; CON: Control; GAPDH: Glyceraldehyde 3-phosphate dehydrogenase.

The protein expression of Ang1 and Ang2 was evaluated by western blot (Figure 6). Compared with CON group, H_2_O_2_ and miR-126 inhibitors decreased the expression of Ang1 (*p* < 0.05) and increased the expression of Ang2 (*p* < 0.05). Compared to H_2_O_2_ group, Ang1 was upregulated in H_2_O_2_+miR-126 mimic group (*p* < 0.05), whereas Ang2 was downregulated (*p* < 0.05). Furthermore, the expression of Ang1 was decreased in H_2_O_2_+miR-126 inhibitor group (*p* < 0.05) but the expression of Ang2 was increased (*p* < 0.05).

### Effect of miR-126 on the expression of proteins associated with PI3K/Akt/GSK3β and ERK1/2 signaling

**FIGURE 7:**
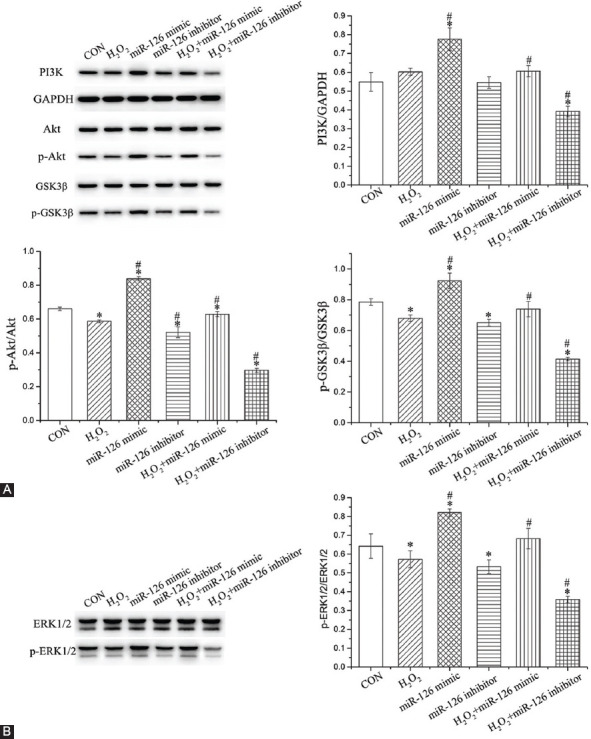
Investigation of signaling pathways involved in miR-126-mediated effects. Expression of proteins associated with (A) PI3K/Akt/GSK3β and (B) ERK1/2 signaling pathway. Data are expressed as the mean ± [SD] (n = 3), *and ^#^represent *p* < 0.05 vs. CON and H_2_O_2_, respectively. PI3K: Phosphatidylinositol 3-kinase; Akt: Protein kinase B; GSK3β: Glycogen synthase kinase 3β; ERK1/2: Extracellular signal-regulated kinase 1/2; CON: Control; GAPDH: Glyceraldehyde 3-phosphate dehydrogenase.

Compared to CON group, the expression of p-Akt, p-GSK3β, and p-ERK1/2 was notably decreased by H_2_O_2_ and miR-126 inhibitors (*p* < 0.05; Figure 7). Compared to H_2_O_2_ group, the expression of PI3K, p-Akt, p-GSK3β, and p-ERK1/2 was increased in H_2_O_2_+miR-126 mimic group (*p* < 0.05) but decreased in H_2_O_2_+miR-126 inhibitor group (*p* < 0.05).

## DISCUSSION

The present study demonstrated that H_2_O_2_ downregulated miR-126 in EPCs and suppressed EPC viability, migration, and tube formation. However, miR-126 overexpression notably improved the biological function of H_2_O_2_-damaged EPCs. Our findings are consistent with the previous reports showing that miR-126 relieved myocardial damage after AMI [35], protected cells from apoptosis, and elevated angiogenesis to prevent myocardial injury [36]. In addition, targeted deletion of miR-126 led to partial embryonic lethality, leaky vessels, and hemorrhaging in mice. This was due to the loss of vascular integrity and impaired endothelial cell migration, proliferation, and angiogenesis [37].

MiR-126 regulates multiple genes and signaling pathways, and its overexpression attenuated vascular endothelial cell apoptosis by activating PI3K/Akt signaling [21]. Upregulation of miR-126 resulted in improved ischemic angiogenesis in mesenchymal stem cells by stimulating Akt/ERK-related signaling [38]. Our study demonstrated that overexpression of miR-126 increased the protein expression of PI3K, Akt, GSK3β, and ERK1/2. The biological function of H_2_O_2_-damaged EPCs, such as cell proliferation, migration, and angiogenesis, was notably improved. In addition, ROS and MDA levels were attenuated, whereas SOD activity was enhanced. It is widely accepted that MDA is involved in the occurrence of oxidative stress [39], while SOD alleviates it [40]. Oxidative stress severely impedes the therapeutic effect of EPC transplantation in myocardial infarction treatment, and miR-126 overexpression inhibits H_2_O_2_-induced oxidative stress in EPCs [10,11]. The PI3K/Akt/GSK3β and ERK1/2 signaling pathways are involved in regulating a variety of cellular processes, such as cell proliferation, apoptosis, and oxidative stress. GSK3β is a downstream signaling molecule of Akt [41], and activation of Akt/GSK3β signaling inhibited oxidative stress and apoptosis in rats with cerebral hypoxic-ischemic injury [42]. Inhibition of MAPK/ERK and PI3K/Akt signaling was previously suggested to suppress angiogenesis in endometrial carcinoma [43]. Additionally, the Akt/GSK3β pathway is involved in the protection against AMI, and its activation stimulated tube formation and accelerated human aortic endothelial cell migration [44]. Consistent with the previous studies, our study showed that miR-126 overexpression promoted EPC proliferation, migration, and tube formation and inhibited H_2_O_2_-stimulated oxidative stress. We hypothesize that the underlying mechanism is associated with the regulation of PI3K/Akt/GSK3β and ERK1/2 signaling.

Ang1 and Ang2 are angiopoietin subtypes that are involved in angiogenesis and vessel maturation. They mainly bind to the endothelial receptor tyrosine kinase Tie-2, which is expressed in endothelial cells and is related to microvascular sprouting and stabilization [45]. Ang1 reportedly played an important role in promoting endothelial cell migration and vessel maturation, while Ang2 is involved in accelerating vascular destabilization and regression [46]. In addition, Ang2 served as an antagonist that inhibited Ang1-induced Tie-2 phosphorylation, thus disrupting angiogenesis [47,48]. According to the previous studies, the ERK1/2 pathway is involved in angiogenesis regulation. Inhibition of ERK1/2 expression reduced angiogenesis in the synovial membrane, which was associated with a decrease in Ang1 and vascular endothelial growth factor expression [49]. Our study suggested that miR-126 overexpression led to the upregulation of Ang1 and downregulation of Ang2, in turn, promoting EPC angiogenesis. These findings are consistent with the results of biological function assays.

## CONCLUSION

Overall, we demonstrated that miR-126 overexpression promoted H_2_O_2_-induced EPC proliferation, migration, and tube formation by regulating PI3K/Akt/GSK3β and ERK1/2 signaling. Our results may contribute to the development of potential therapeutic strategies against AMI. However, the study was designed and conducted only at the cellular level. Further in-depth experiments will be performed in animals to verify our conclusions. In addition, specific target genes of miR-126 will be investigated in prospective studies.
